# Global research hotspots and trends in constraint-induced movement therapy in rehabilitation over the past 30 years: a bibliometric and visualization study

**DOI:** 10.3389/fneur.2024.1375855

**Published:** 2024-06-14

**Authors:** Jie Xu, Meng Chen, Xin Wang, Zijuan Cai, Yanjie Wang, Xiaobing Luo

**Affiliations:** ^1^Department of Sports Medicine, Sichuan Provincial Orthopedics Hospital, Chengdu, China; ^2^Department of Emergency Medicine, Nanchong Hospital of Traditional Chinese Medicine, Nanchong, China; ^3^Health Science Center, Peking University, Beijing, China; ^4^College of Physical Education and Health, Geely University of China, Chengdu, China

**Keywords:** CIMT, constraint-induced movement therapy, stroke, exercise rehabilitation, Citespace, bibliometrics

## Abstract

**Background:**

Stroke is a cerebrovascular disease with high prevalence and mortality, and upper limb hemiparesis is a major factor limiting functional recovery in stroke patients. Improvement of motor function in stroke patients through various forms of constraint-induced movement therapy (CITM) has been recognized as safe and effective in recent years. This research field lacks a comprehensive systematic and clear vein combing analysis, analyzing the literature research of CIMT in the field of rehabilitation in the past three decades, summarizing the research hotspots and cutting-edge trends in this field, in an effort to offer ideas and references for subsequent researchers.

**Methods:**

Relevant literature on CIMT in rehabilitation was collected from 1996 to 2024 within the Web of Science database’s core dataset by using CiteSpace6.1, VOSviewer1.6.18, R-bibliometrix4.6.1, Pajek5.16, Scimago Graphica 1.0.26 software for visualization and analysis.

**Results:**

There were 970 papers in all United States was ranked first with 401 papers. Alabama Univ was ranked first for institutions with 53 papers. Neurorehabilitation and Neural Repair was ranked first for journals with 78 papers, and Taub E was ranked first for author publications with 64 papers. Research keywords were CIMT, stroke rehabilitation, upper extremity function, lower extremity gait balance, randomized controlled trials, physical therapy techniques (transcranial magnetic stimulation and sensory amplitude electrical stimulation), primary motor cortex plasticity, lateral dominance (spatial behaviors), cerebral vascular accidents, activities of daily living, hand function, disability, functional restoration, bimanual training, aphasia, acquired invalidity, type A Botulinum toxin and joystick riding toys.

**Conclusion:**

The current state of research shows that CIMT still has a vast potential for development in the field of rehabilitation research. The research hotspots are the clinical efficacy of CIMT combined with other therapies (botulinum toxin type A, transcranial direct current stimulation, virtual reality, mirror therapy, robotic-assisted) to enhance the functionality of upper limb hemiparesis in stroke patients, the mechanism of CIMT to improve the plasticity of the motor cortex through electrophysiological and imaging methods, and improvement of lower limb gait balance function in stroke patients and aphasia applications, the optimal intervention time and dose, and exploration of CIMT in new settings such as robot-assisted, telemedicine, and home rehabilitation.

## 1 Introduction

Strokes are the second leading cause of death globally and the third leading cause of disability (1). It is estimated that strokes result in 5.5 million deaths each year, with up to 50% of survivors experiencing long-term disability (2). Around 65% of patients are unable to use their affected hand for daily activities 6 months post-stroke, and up to 35% of individuals with lower limb paralysis do not fully regain their physical abilities (3), and upper extremity hemiparesis is a major limiting factor in functional recovery. When stroke patients fail to effectively use their affected upper extremity, they may develop ‘learned nonuse, ‘where they rely on their unaffected side for daily tasks (4). Constraint-induced movement therapy (CIMT) can enhance upper extremity function by addressing learned disuse and leveraging use-dependent neuroplasticity (5). CIMT comprises reinforcement training, shaping training, ‘transfer kits’ to facilitate the application of treatment benefits to daily activities, and discouragement of compensatory strategies (6, 7). During the 2 weeks of the program, the less affected upper extremity is immobilized in a sling, and 90% of waking hours (including time spent receiving direct treatment) are spent wearing a splint, glove, or cast, while the more severe upper extremity receives 6 h of treatment per day following a routine procedure (8, 9). Each session includes shaping and targeted, task-specific exercises that increase in difficulty as function or performance improves. Traditional CIMT is most effective for individuals with specific criteria, including a minimum of 10° of wrist and thumb extension, extension of at least 2 other fingers in the most impaired upper extremity, and the ability to stand and transfer independently (8, 10). Challenges have been reported by both patients and therapists in implementing this approach in clinical settings (11), leading to the development of distributed or modified forms of CIMT (dCIMT/mCIMT). These modified forms reduce the duration of each training session (0.5–3 h/session) and daily time commitment (5–9 h/day), extending the overall duration of treatment (3–10 weeks). Evidence suggests that both mCIMT and dCIMT improve motor function in affected limbs (12–14). In addition to improving post-stroke hemiparesis, CIMT also addresses related issues such as unilateral neglect (15, 16) and aphasia (6). CIMT has shown effectiveness in treating Parkinson’s patients (17), children with cerebral palsy (18), and improving fine and gross motor skills, daily living abilities, and cognitive function in the upper limbs. Furthermore, CIMT has shown promise in improving conditions related to peripheral nerve diseases, such as brachial plexus nerve injury (19, 20) and finger dystonia (21).

Over the past three decades, clinical studies on CIMT/mCIMT to enhance stroke patients’ quality of life and function have significantly advanced. Although the efficacy of CIMT/mCIMT has reached a consensus, the mechanism of its action remains unclear. Further research is needed to verify issues such as the intensity of training, timing of intervention, and duration of treatment efficacy. The treatment of stroke and other mental illnesses faces new opportunities and challenges due to the development of new ideas and technologies. Investigating CIMT research trends and hotspots in the rehabilitation sector is crucial, yet there is a lack of corresponding bibliometric studies in this field. Therefore, this study aims to analyze hotspots, frontiers, and development trajectories to provide a fresh perspective for a comprehensive understanding of the area and offer researchers valuable references and support.

## 2 Methodology

### 2.1 Search strategy and data retrieval

**Figure 1:**
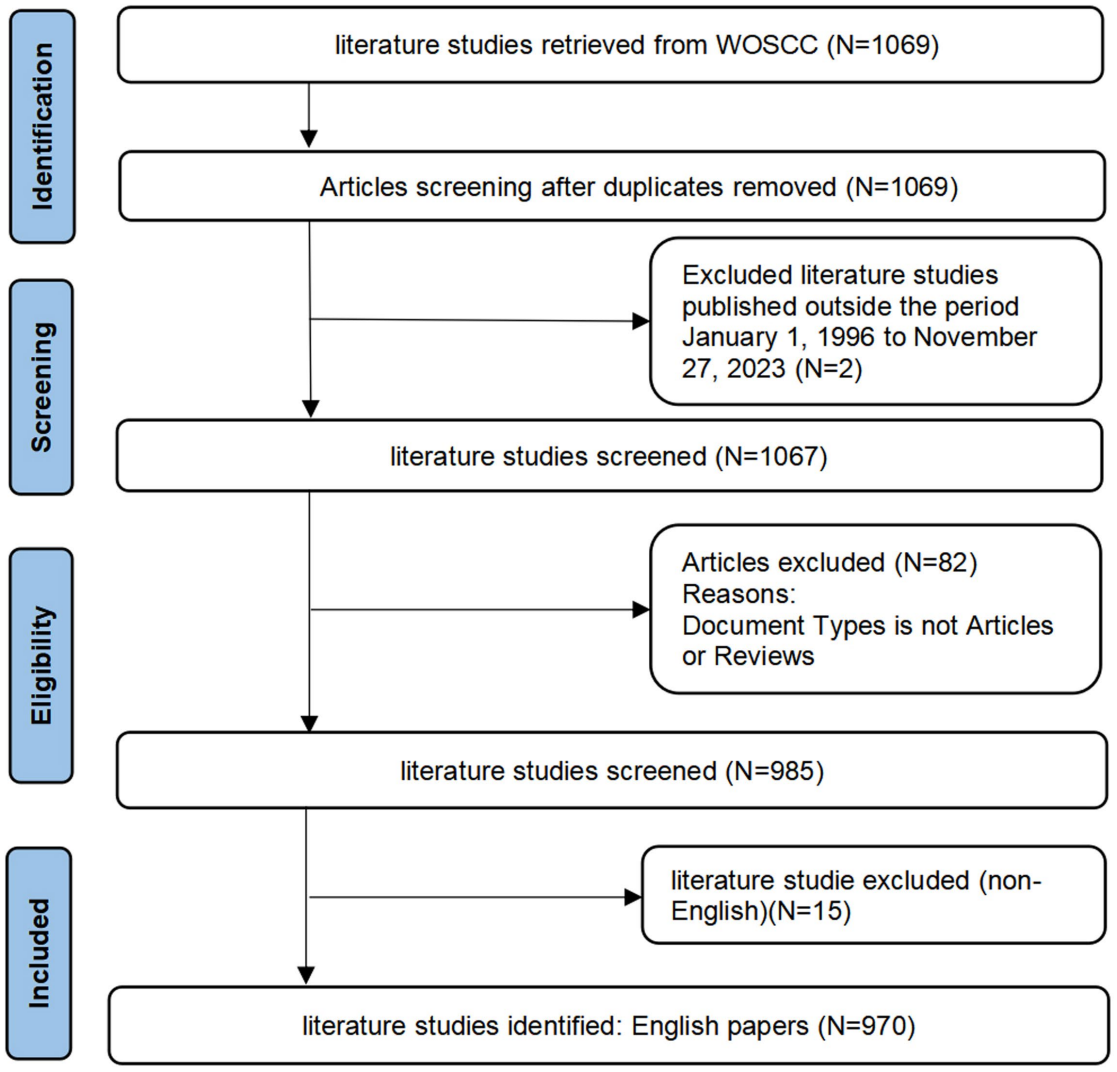
Workflow diagram.

Bibliometric research is a quantitative analysis of scientific literature. Gathered data from Web of Science in “plain text,” including “complete records and references.” Collected data (500 records per file) was exported, renamed “download,” and input into Citespace software. Citespace software visualizes scientific literature, generating maps to understand patterns. Drew visualization maps by selecting specific node types, aiding comprehension of scientific literature patterns. Supplementary Table S1 provides detailed information on the search strategy. The selection of the literature for this study was done using the following inclusion and exclusion standards: The literature met the following requirements: (i) it was published between January 1, 1996, and January 1, 2024; (ii) it was written in English; (iii) it was an article or review; (iv) it did not place restrictions on the species or organisms it studied; and (v) duplicates were eliminated from the analysis of the publications to guarantee the dataset’s uniqueness. Since the data in the publications contained no personally identifiable information about the patients, ethical approval was not necessary. In Figure 1, the relevant workflow diagram is displayed.

### 2.2 Literature screening

Independently, two evaluators perused the literature. The article’s title and abstract served as the basis for the first screening. Next, the inclusion and exclusion criteria were used to determine which papers should be included or excluded. If there was any doubt, a third assessor, who possessed veto power, read the entire document and made the final decision.

### 2.3 Methods of statistical analysis

A number of tools, including CiteSpace version 6.1.R6, R-Studio based R-bibliometrix version 4.6.1, Pajek version 5.16, Scimago Graphica version 1.0.26, and VOSviewer version 1.6.18, were used in our bibliometric analysis research.

CiteSpace version 6.1.R6 (Drexel University, Philadelphia, PA) is a software tool developed by Prof. Chen C. It excels in identifying research hotspots and trends within the academic field by analyzing citation relationships and evolutionary trends in the literature, which allows for the discovery of key research themes and widely cited papers. In this study, we do a visual analysis using CiteSpace that covers several features, including journal citations, reference analysis, country and institutional distribution, and keyword and citation bursts. The network structure has been simplified and key aspects have been highlighted by selecting “Pruning sliced networks” and “Pathfinder,” and the filtering criteria has been set to “Top N,” the threshold is set to 50, and the time scale is set to 1 year. See Supplementary Table S2 for details.

VOSviewer version 1.6.18 (Leiden University, Leiden, the Netherlands) is a visualization software developed by Prof. Van Eck and Prof. Waltman, which focuses on co-word relationships between the literature and the analysis of co-word frequencies. It can help researchers to discover common themes and concepts in the literature and to construct co-occurrence network diagrams for visualization. Analyzing the co-occurrence frequency and temporal information of keywords in the literature reveals the evolution process of research topics and the development trend of keywords and helps researchers understand the development dynamics of academic fields (22). In this work, VOSviewer was utilized to investigate keyword co-occurrence and coverage networks as well as to analyze and display the distribution of nations, institutions, authors, and co-cited authors. The detailed parameters were: the keyword inclusion criterion was a minimum number of occurrences of 5, the association strength method was chosen for the normalization process, the cluster resolution and minimum cluster size were both 1 and merge small clusters were checked, the minimum strength of cluster links was 0, and the maximum number of links was 1,000, as detailed in Supplementary Table S3.

R-Studio-based R-bibliometrics version 4.6.1, Pajek version 5.16, and Scimago Graphics version 1.0.26 are powerful tools for multi-modal and multi-dimensional geo-visualization. In this study, it was used to highlight the inter-cooperative network relationships between different countries or regions.

## 3 Results

### 3.1 Characteristics and trends in the volume of publications

**Figure 2:**
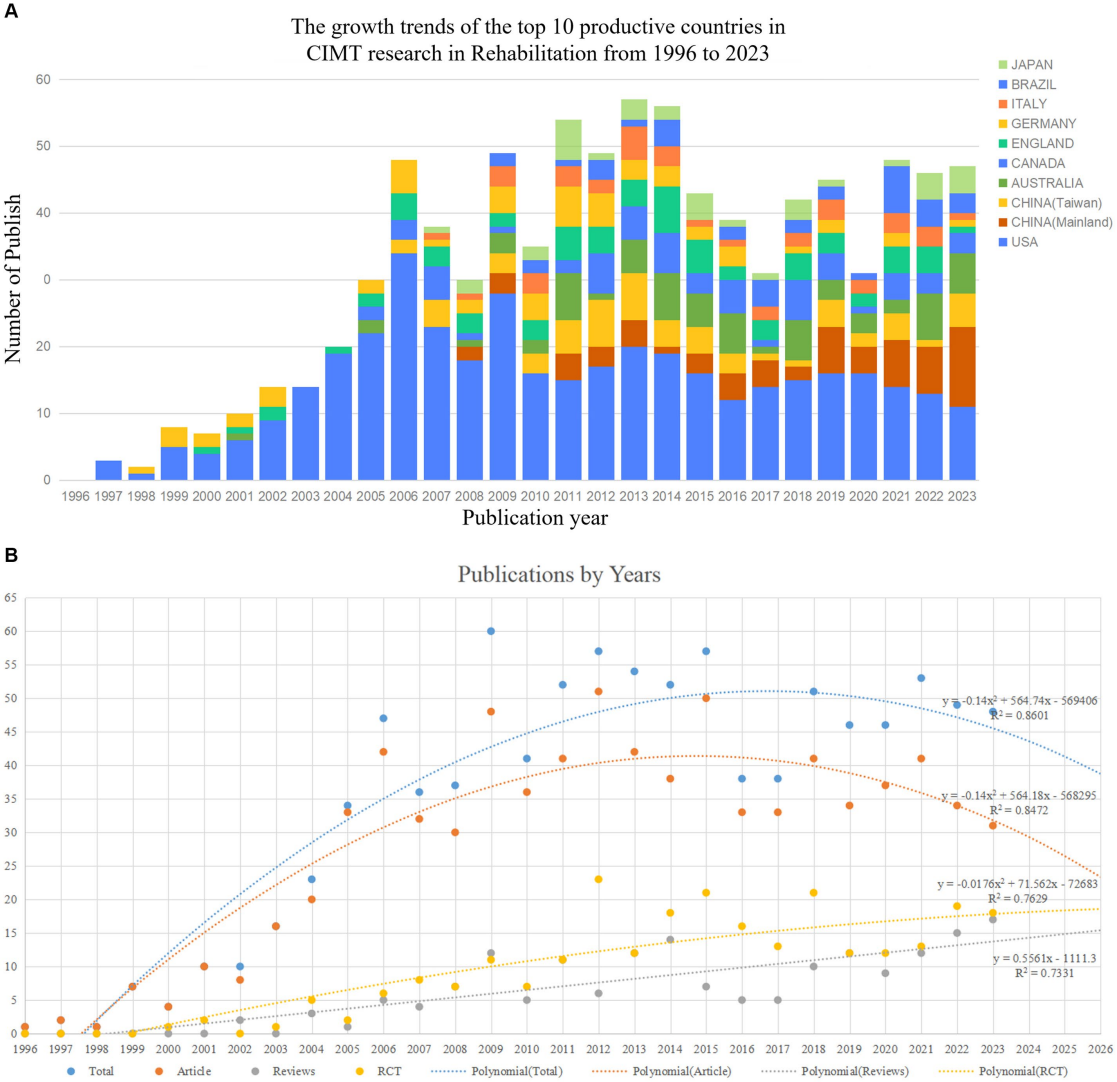
**(A)** Bibliometric analysis of WoS core database output. **(B)** Trends in publications and corresponding polynomial fit curves.

By searching the core database of Web of Science, a total of 1,069 CIMT in the field of rehabilitation research-related literature was retrieved, excluding the literature publication time not from January 1, 1996, to January 01, 2024, 2 literature, then excluding the literature type for non-article and review of 82 literature, and finally excluding non-English literature 15, and finally 970 literatures were included, as shown in Figure 1. Of them, 796 comprised 82.1% of original research publications, while 174 reviewed papers made up 17.9%. The total citation frequency was 46,987 times, and the average citation frequency per paper was 48.44 times. The annual publishing volume across several nations is displayed in Figure 2A. From 1996 to 2024, the quantity of research articles in the topic of rehabilitation in CIMT remained rather consistent, showing remarkable expansion in 2006 as the number of publications rose by 38.2% over the year before, the annual number of publications was maintained at around 48 and peaked at 60 in 2009. In general, the top three countries for research in the sector continue to be the US, China, and Canada. China has able to surpass the US as the leading nation in terms of yearly publications from 2023. The U.S. has the highest share in terms of total publications, with a tendency to be overtaken by China in terms of annual publications. Through an investigation of polynomial fits between the number of publications and the year of publication, we found a significant correlation (for total papers, articles, reviews, and RCTs, the coefficients of determination (R2) were 0.8601, 0.8472, 0.7629, and 0.7331, in that order). We predicted that the number of publications in 2026 would reach about 39, including about 23 original articles, 16 reviews, and 19 RCTs, as shown in Figure 2B. However, it can be observed that there is still a relative lack of high-quality RCT studies despite the increasing number of publications year by year.

### 3.2 Countries or regions collaboration analysis

**Figure 3:**
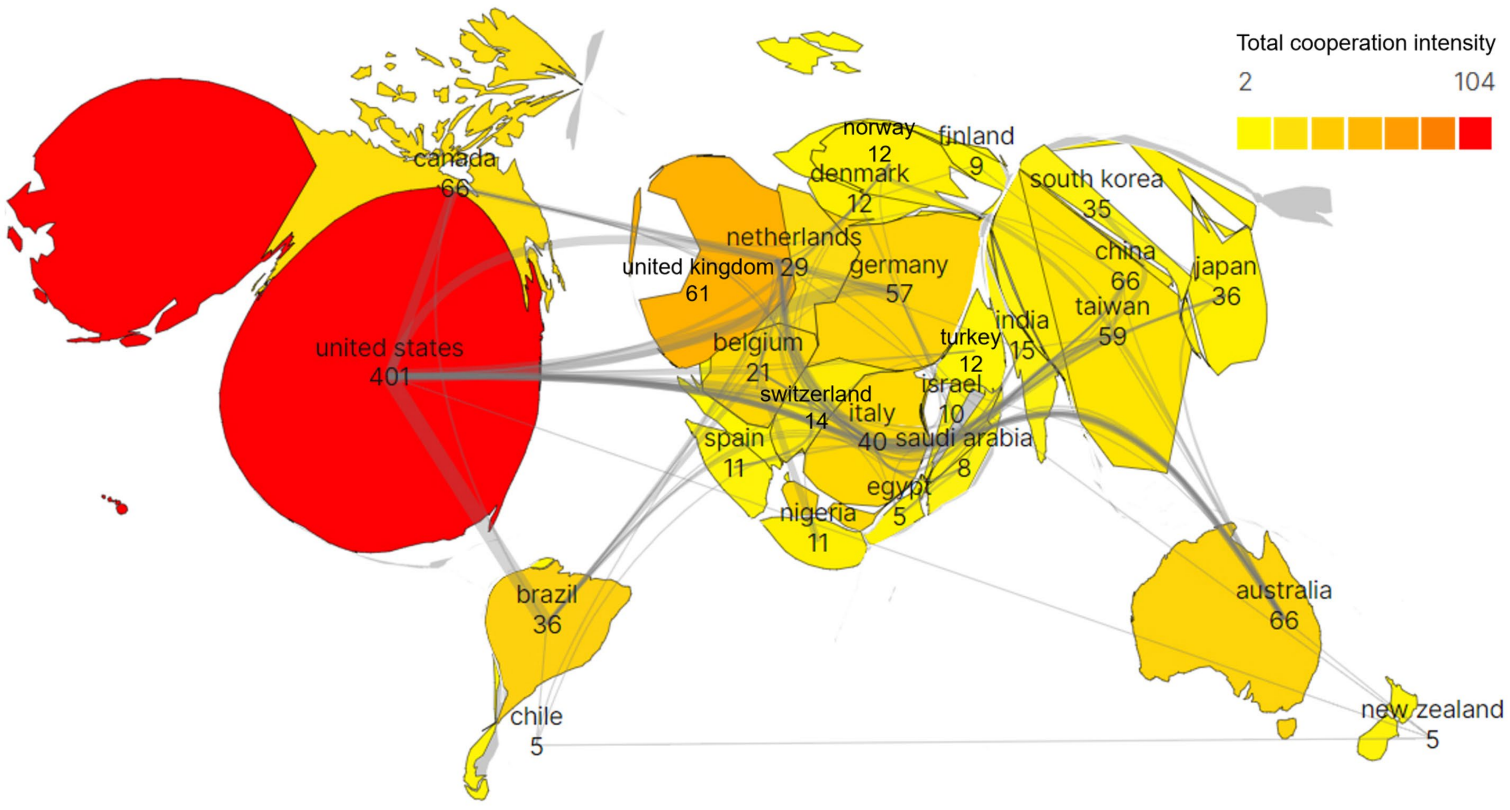
Populated chart of CIMT’s bibliometric analysis of country collaborations in the field of rehabilitation.

The resultant graphic includes 58 nations or areas in total, of which 10 have at least 30 articles. The data pertaining to the top 10 nations in terms of publications is displayed in Table 1. In the filled Figure 3, the larger percentage of the country area represents more articles sent, the color is darker, which represents a stronger cooperation relationship, the thickness of the lines indicates the intensity of the link, while the connecting lines show the level of collaboration. Therefore, it can be inferred that the United States (401 articles or 41.34%) is the most active country, followed by China (125 articles or 12.89%, which can be categorized into 66 articles or 6.80% in Mainland China and 59 articles or 6.08% in Taiwan, China) and Australia (66 articles or 6.80%). These three nations are the biggest contributors to the area, with a combined total of publications that account for 61% of all publications. In terms of total citation rate (TC), total link strength (TLS), and H-index, the US leads the world, while the United Kingdom leads in average citations per publication (ACPP). The TLS indicates the level of partnerships between researchers, while the H-index is frequently employed to gauge academic influence.

**Table 1:** Top 10 countries with high impact of CIMT research in the field of rehabilitation.

Country	NP	TC	ACPP	TLS	H-index
United States	401 (41.34%)	26,010	64.86	6,566	77
China (Mainland)	66 (6.80%)	938	14.21	1,196	17
Australia	66 (6.80%)	5,146	77.97	1,606	23
Canada	66 (6.80%)	2,816	42.67	1,246	24
England	61 (6.29%)	6,928	113.57	1,461	30
China (Taiwan)	59 (6.08%)	1746	29.59	1,474	26
Germany	57 (5.88%)	5,804	101.82	1782	30
Italy	40 (4.12%)	1,439	35.98	1,126	15
Brazil	36 (3.71%)	792	22.00	839	14
Japan	36 (3.71%)	656	18.22	547	11

NP, number of publications; TC, total citation; TLS, total link strength; ACPP, average citations per publication.

### 3.3 Research institution collaboration analysis

**Figure 4:**
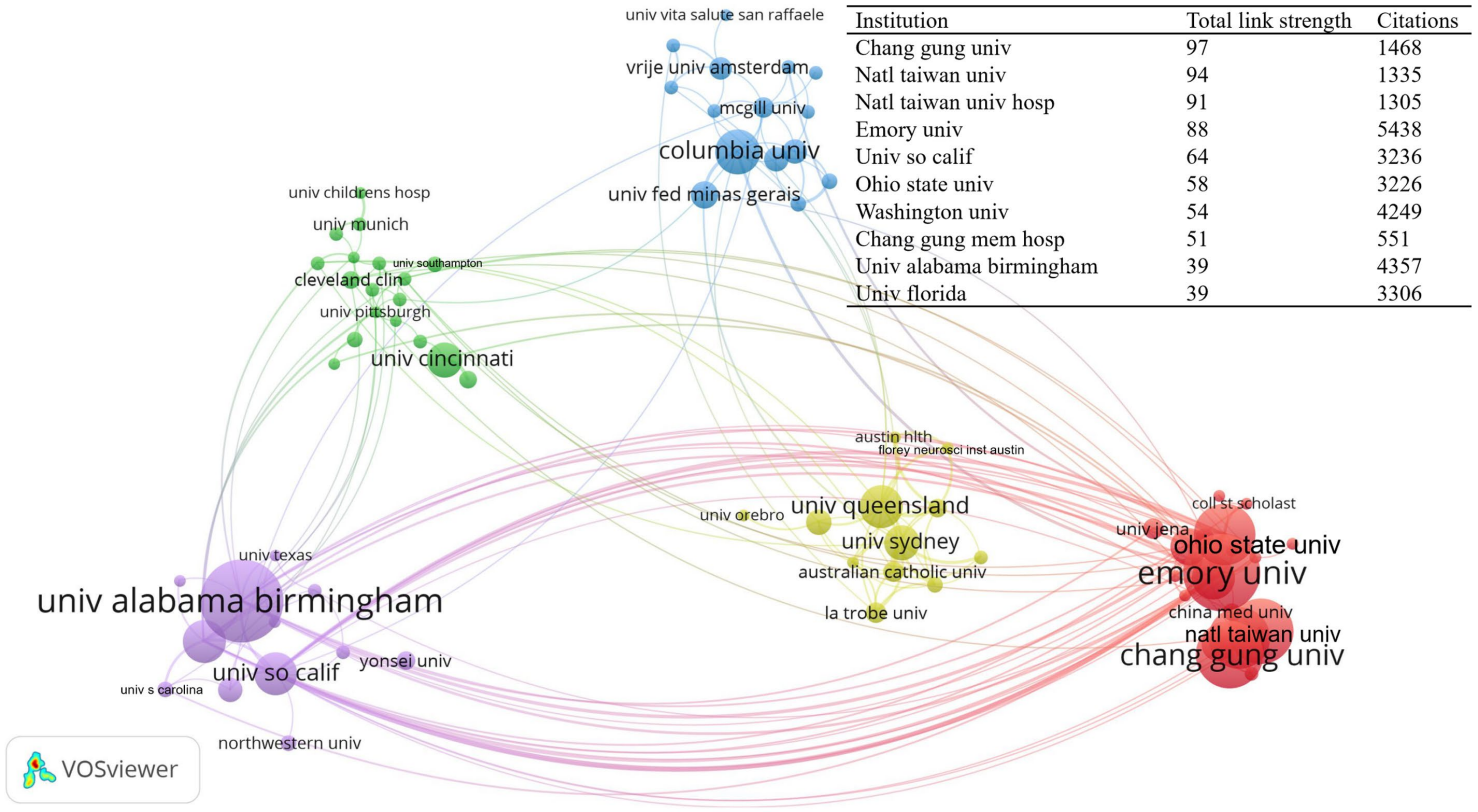
CIMT’s intensity of collaboration across agencies in the rehabilitation field, http://tinyurl.com/ynp5b7fb.

In the realm of rehabilitation, 1,377 institutions released studies connected to CIMT, of which 28 institutions had ≥10 publications. Table 2 displays data on the top ten institutions based on the quantity of publications, and 36.39% of the total number of articles came from the top 10 universities with the most publications. Figure 4 illustrates the international collaborations between the 95 institutions that have collaborated on at least five publications. It is noteworthy that the University of Alabama Birmingham (United States) performed most prominently with 53 publications or 5.46% of all publications, followed by Emory Univ (United States) (49 publications, 5.05%) and Natl Taiwan Univ (China) (40 publications, 4.12%). It is noteworthy that among universities, Emory University has the greatest TC and TLS. University Florida and University Alabama Birmingham had the greatest ACPP and H-index, respectively. Chang Gung University, Emory University, and University of Alabama in Birmingham are three universities that have improved their research partnerships with a few other universities.

**Table 2:** CIMT top 10 high impact institutions in the field of rehabilitation.

Institution	NP	TC	ACPP	TLS	H-index	Location
Alabama Univ	53 (5.46%)	4,537	85.60	2,161	39	United States
Emory Univ	49 (5.05%)	5,438	110.98	2,576	29	United States
Natl Taiwan Univ	40 (4.12%)	1,335	33.38	1,603	22	China (Taiwan)
Chang Gung Univ	40 (4.12%)	1,468	36.70	1,625	23	China (Taiwan)
Natl Taiwan Univ Hosp	37 (3.81%)	1,305	35.27	1,485	20	China (Taiwan)
Ohio State Univ	37 (3.81%)	3,226	87.19	1736	30	United States
Columbia Univ	25 (2.58%)	1988	79.52	938	11	United States
Univ Florida	24 (2.47%)	3,306	137.75	990	16	United States
Univ Queensland	24 (2.47%)	1,387	57.79	873	15	Australia
Univ So Calif	24 (2.47%)	3,236	134.83	1,517	22	United States

### 3.4 High-impact authors collaboration analysis

**Figure 5:**
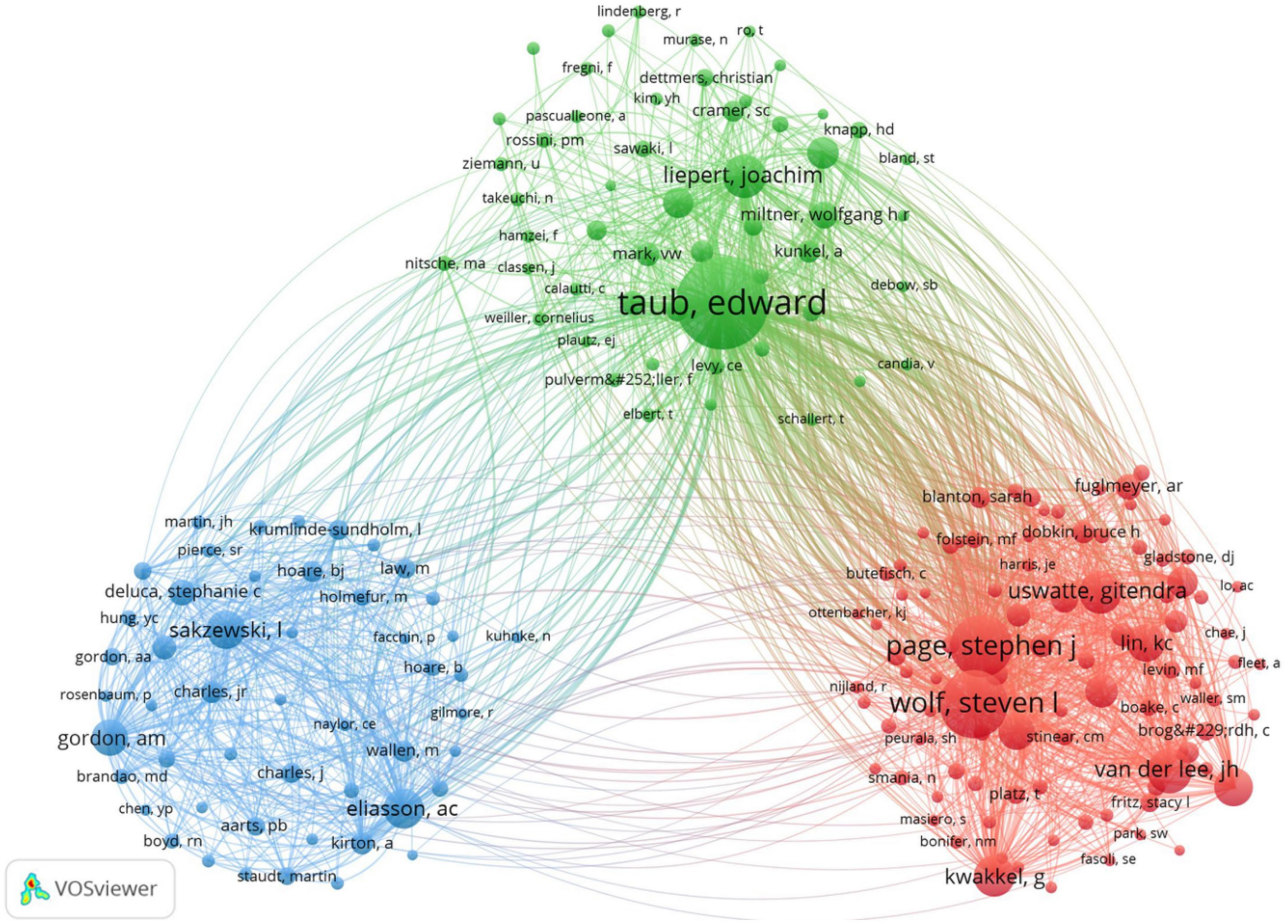
Visualization of author co-citation analysis generated based on VOSviewer software. Each node represents an author, and the size of each circle is determined by the author’s co-citation, https://tinyurl.com/yppfvox9.

Of the 3,444 authors included in the graphic map, 91 had ≥5 publications. Table 3 displays the information of the top ten academics in regards to quantity of publications and citations. Three of the most prominent researchers of the CIMT in the field of rehabilitation, i.e., Taub E, Lin K, and Uswatte G, with 64 (6.60%), 43 (4.43%) and 40 (4.12%) publications, respectively, are very active and influential authors. TC, ACPP, H-index, and TLS of Taub E, University of Alabama, United States were ranked top. The top three authors of CIMT with regard to of co-citation frequency in the field of rehabilitation were Taub E. (1806), Wolf SL. (976), and Page S. J. (818), according to the author co-citation study carried out by VOSviewer. The network of co-cited author relationships is depicted in Figure 5, with each node’s size according to how frequently it has been mentioned. Various colors show various clusters, which comprise 196 authors with a citation frequency of 30 or more. The connection and thickness between the nodes, respectively, reflect the co-citation relationship and its strength.

**Table 3:** CIMT’s top 10 high impact authors in the field of rehabilitation.

Author	NP	TC	ACPP	TLS	H-index	Institution
Taub E	64 (6.60%)	2,629	41.08	132	39	University of Alabama
Uswatte G	43 (4.43%)	1,512	35.16	121	29	University of Alabama
Wolf SL	40 (4.12%)	1,233	30.83	69	28	Emory University
Lin KC	31 (3.20%)	582	18.77	73	22	Natl Taiwan Univ
Wu CY	29 (2.99%)	592	20.41	75	22	Chang Gung Univ
Page SJ	29 (2.99%)	656	22.62	16	19	University of Cincinnati
Morris DM	26 (2.68%)	195	7.50	45	10	University of Alabama
Mark VW	22 (2.27%)	363	16.50	61	16	University of Alabama
Blanton S	20 (2.06%)	438	21.90	54	14	Emory University
Levine P	19 (1.96%)	612	32.21	24	17	University of Cincinnati

### 3.5 High-impact journals collaboration analysis

**Figure 6:**
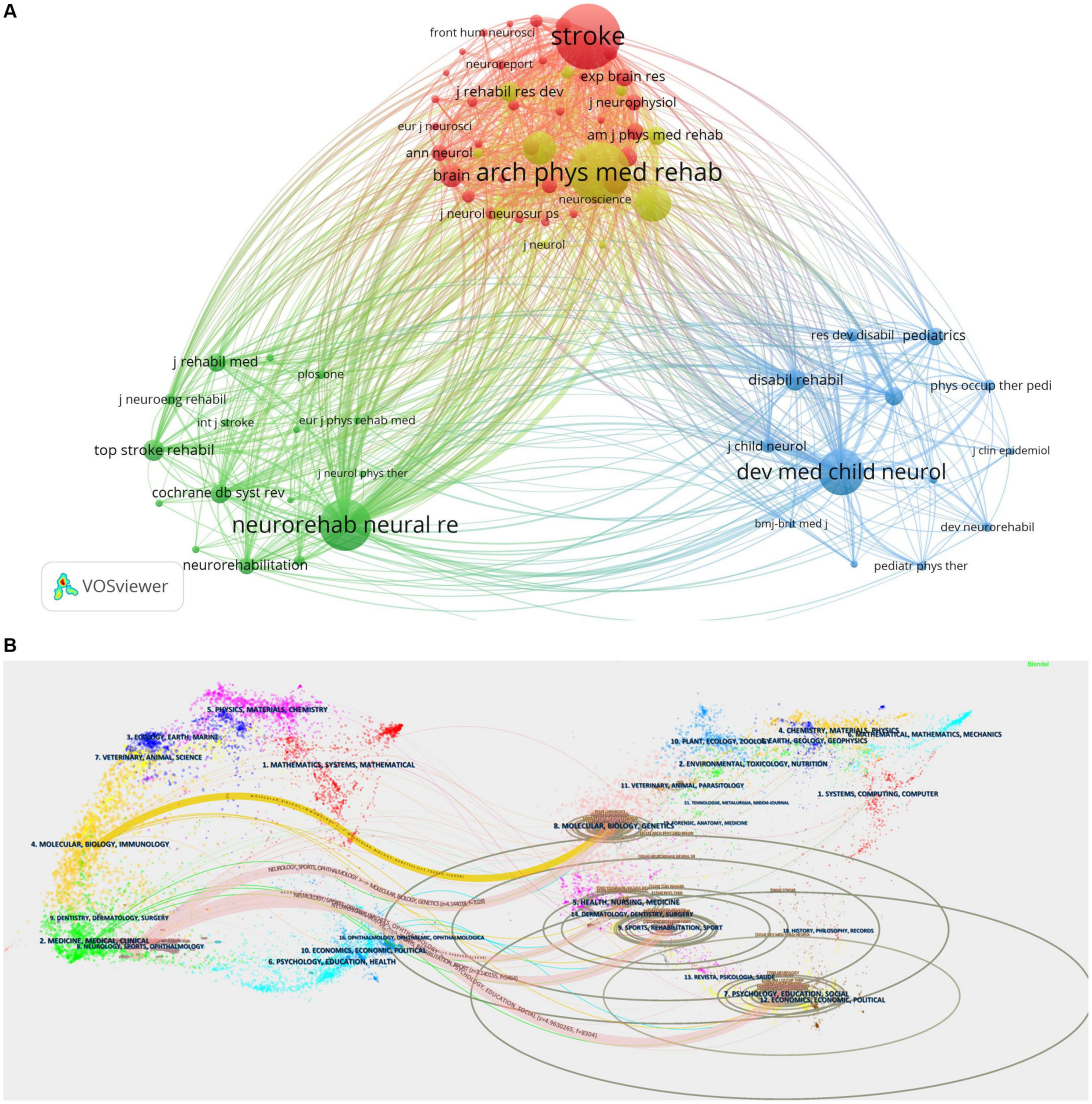
**(A)** Visualization of journal co-citation analysis generated based on VOSviewer software, https://tinyurl.com/yord8ryt. **(B)** CIMT double plot overlay of citing and cited journals in the field of rehabilitation. Cited journals are on the left, cited journals are on the right, and the connecting lines represent citations.

The 261 journals that contained the 970 documents that were retrieved had the highest number of publications across the top three journals: Archives of Physical Medicine and Rehabilitation (63 articles with 1,275 total citations and 20.24 average citations), Neurorehabilitation and Neural Repair (78 articles with 1,392 total citations and an average of 17.85 citations), and Physical Therapy (33 articles with 411 total citations and 12.45 average citations). The top three journals in this field of research with high H-index and top three H-index are Archives of Physical Medicine and Rehabilitation (40), Neurorehabilitation and Neural Repair (37), and Stroke (25). There is a good amount of research in the relevant domains, as indicated by the Q1 and Q2 classifications assigned to all 10 journals. The fact that these 10 journals originated from Europe and the United States suggests that they have played a crucial role in advancing scholarship in the field, and according to the VOSviewer-generated journal co-citation analysis, the top three co-cited journals were Stroke (3,871 times), Archives of Physical Medicine and Rehabilitation (3,511 times), and Neurorehabilitation and Neural Repair (2,654 times). Table 4 and Figure 6A show the network of co-cited journal relationships, which includes 75 journals with a citation frequency of 100 or more times. Presently, journals publishing publications in this field of study have a significant impact. The double graph overlay’s colored pathways connecting journal clusters show the citation linkages between the citing and cited journals. The colored paths indicate that studies published in NEUROLOGY, SPORTS, OPHTHALMOLOGY journals usually cite studies published in SPORTS, REHABILITATION, SPORT and PSYCHOLOGY, EDUCATION, SOCIAL. Figure 6B provides further details regarding the typical citing and referenced journals in each cluster. For example, the most representative journals in the Physical Education/Rehabilitation/Sports cluster are Archives of Physical Medicine and Rehabilitation, Neurorehabilitation and Neural Repair, Clinical Rehabilitation, and Physical Therapy. The journals most represented in the Psychology/Education/Sociology group are Stroke, Developmental Medicine and Child Neurology, and Neurology.

**Table 4:** Status of CIMT high volume journals in the field of rehabilitation.

Journal	NP	TC	ACPP	TLS	H-index	IF (2023)	JCR (2023)
Neurorehabilitation and Neural Repair	78	1,392	17.85	1,309	37	4.1	Q1
Archives of Physical Medicine and Rehabilitation	63	1,275	20.24	1,228	40	4.3	Q1
Physical Therapy	33	411	12.45	563	21	3.8	Q1
Neurorehabilitation	33	244	7.39	486	14	2	Q2
Clinical Rehabilitation	33	481	14.58	687	18	3	Q1
Stroke	31	1,332	42.97	1,001	25	8.4	Q1
Topics in Stroke Rehabilitation	27	184	6.81	359	16	2.2	Q2
American Journal of Occupational Therapy	27	166	6.15	366	13	2.9	Q1
Disability and Rehabilitation	21	111	5.29	255	13	2.2	Q2
Frontiers in Neurology	20	31	1.55	251	8	3.4	Q2

## 4 Keyword visualization and analysis

**Figure 7:**
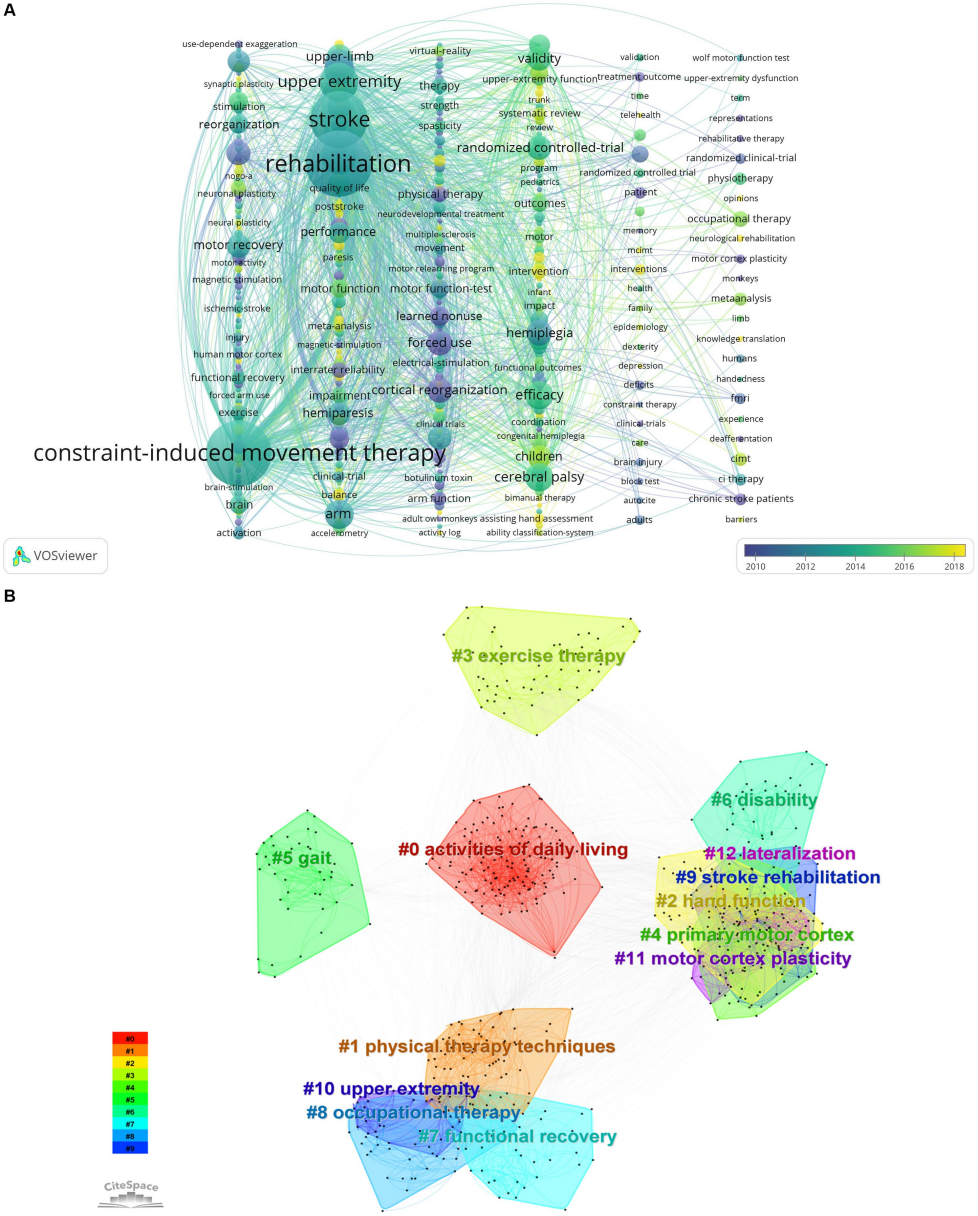

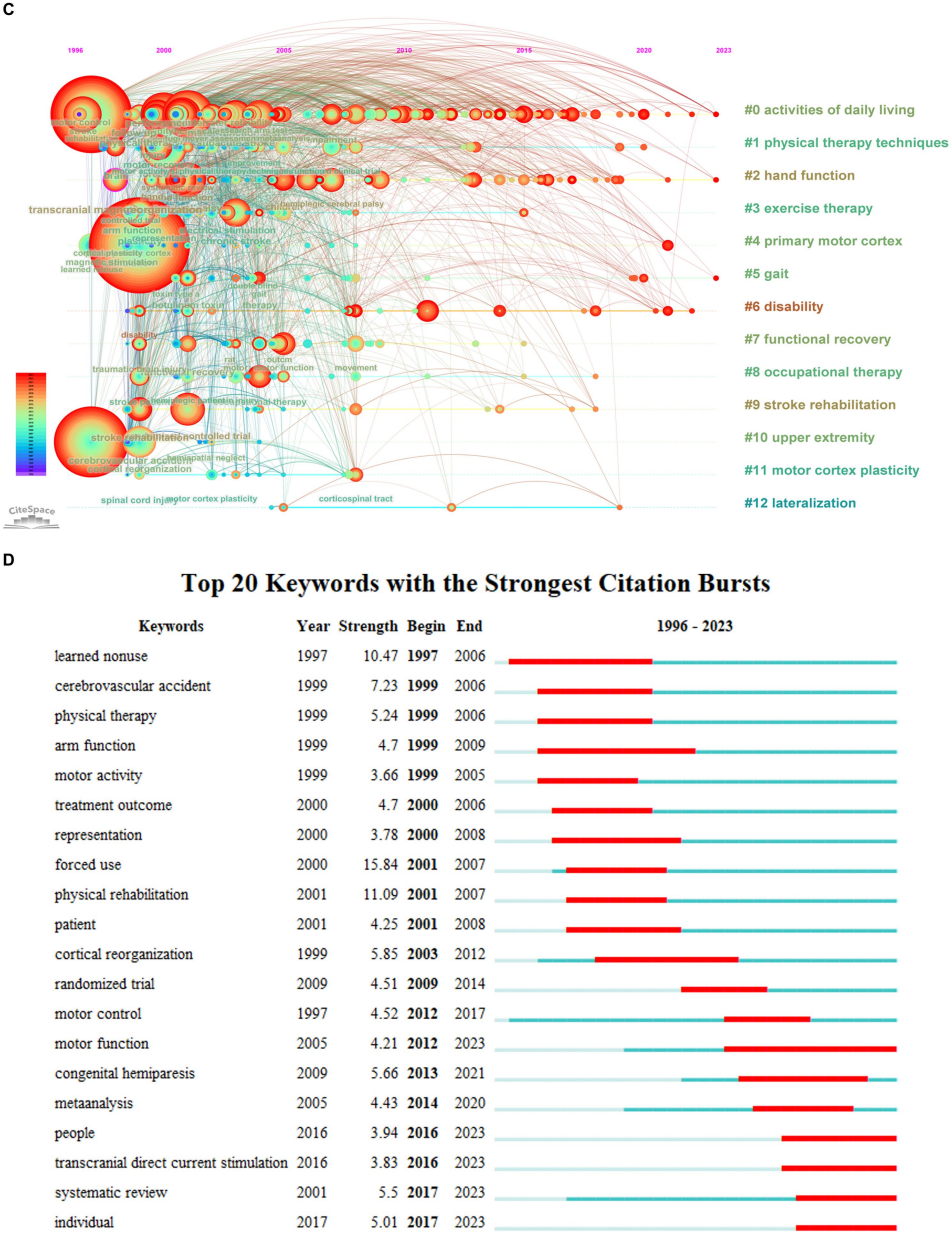
**(A)** Keyword time view of CIMT in rehabilitation based on VOSviewer, http://tinyurl.com/ysc66zbg. **(B)** Citespce-based keyword co-occurrence clustering of CIMT in the field of rehabilitation. **(C)** Timeline view of CIMT keywords in the field of rehabilitation. **(D)** Keyword bursting map for CIMT in the field of rehabilitation.

When it comes to examining research trends and summarizing research hotspots, keyword analysis is crucial. Using the keyword time view produced by VOSviewer, each vertical bar in Figure 7A represents a cluster, and the circle represents the keyword, with a larger circle indicating a higher frequency of occurrence of the term. The connecting lines show the connections between terms, and the skewed yellow node color represents the years from far to near. The keywords with high centrality and frequency in Table 5 represent the research hotspots in that period of time. The keyword co-occurrence clustering map in this field is shown in Figure 7B. Thirteen clusters in all were created using the conventional log-likelihood rate (LLR) technique, and via analysis of the keyword clusters, it was shown that the homogeneity of the study improved with increasing degree of aggregation (23). The biggest cluster is denoted by #0, and so on. The cluster number is inversely proportionate to the cluster size. Keyword co-occurrence and cluster analysis yielded activities of daily living, physical therapy techniques, hand function, motor therapy, primary motor cortex, gait, disability, functional recovery, occupational therapy, stroke rehabilitation, upper extremity, motor cortex plasticity, and lateral dominance as the current research hotspots. Figure 7C Timeline view of CIMT’s keywords in the field of rehabilitation, it can be concluded that research hotspots such as #0 activities of daily living, #2 hand function, #5 gait, and #6 disability have been continuing to this day and will remain so. Furthermore, as Figure 7D illustrates, the top 20 terms with the strongest burst were chosen by extending the burst period to 2 years. The symbol “Strength” in the graphic denotes the burst’s size, while “Begin” and “End” signify its start and end times, respectively. The time interval is represented by the blue line, while the burst’s length is shown by the red line. By analyzing the burst words, we may investigate this field’s projected development tendencies and hotspots for study, especially when analyzing the keywords with the duration of the burst time up to now, which is of important reference value and guidance significance. The most frequent mutation intensity is “forced use” (15.84), followed by “physical rehabilitation” (11.09), and the third one is “learned nonuse” (10.09). learned nonuse” (10.47). The most frequent words were “motor function,” “people,” “transcranial direct current stimulation,” “transcranial current stimulation,” “physical rehabilitation,” and “learned nonuse” (10.47). “motor function,” “people,” “transcranial direct current stimulation,” “systematic review,” and “individual” have all persisted so far, and are probably going to stay hot.

**Table 5:** CIMT’s high-frequency keywords and centrality in the field of rehabilitation top 10.

Keyword	Frequency	Keyword	Centrality
Constraint-induced movement therapy	618	Transcranial magnetic stimulation	0.11
Rehabilitation	438	Functional recovery	0.1
Upper extremity	372	Cerebral palsy	0.09
Upper extremity function	195	Cerebrovascular accident	0.09
Reliability	170	Chronic stroke	0.08
Cerebral palsy	145	Reorganization	0.08
Forced use	144	Asymmetry	0.08
Stroke	120	Motor function test	0.07
Efficacy	116	Stroke rehabilitation	0.07
Randomized controlled trial	109	Brain	0.07

## 5 Visual analysis of key literature

**Figure 8:**
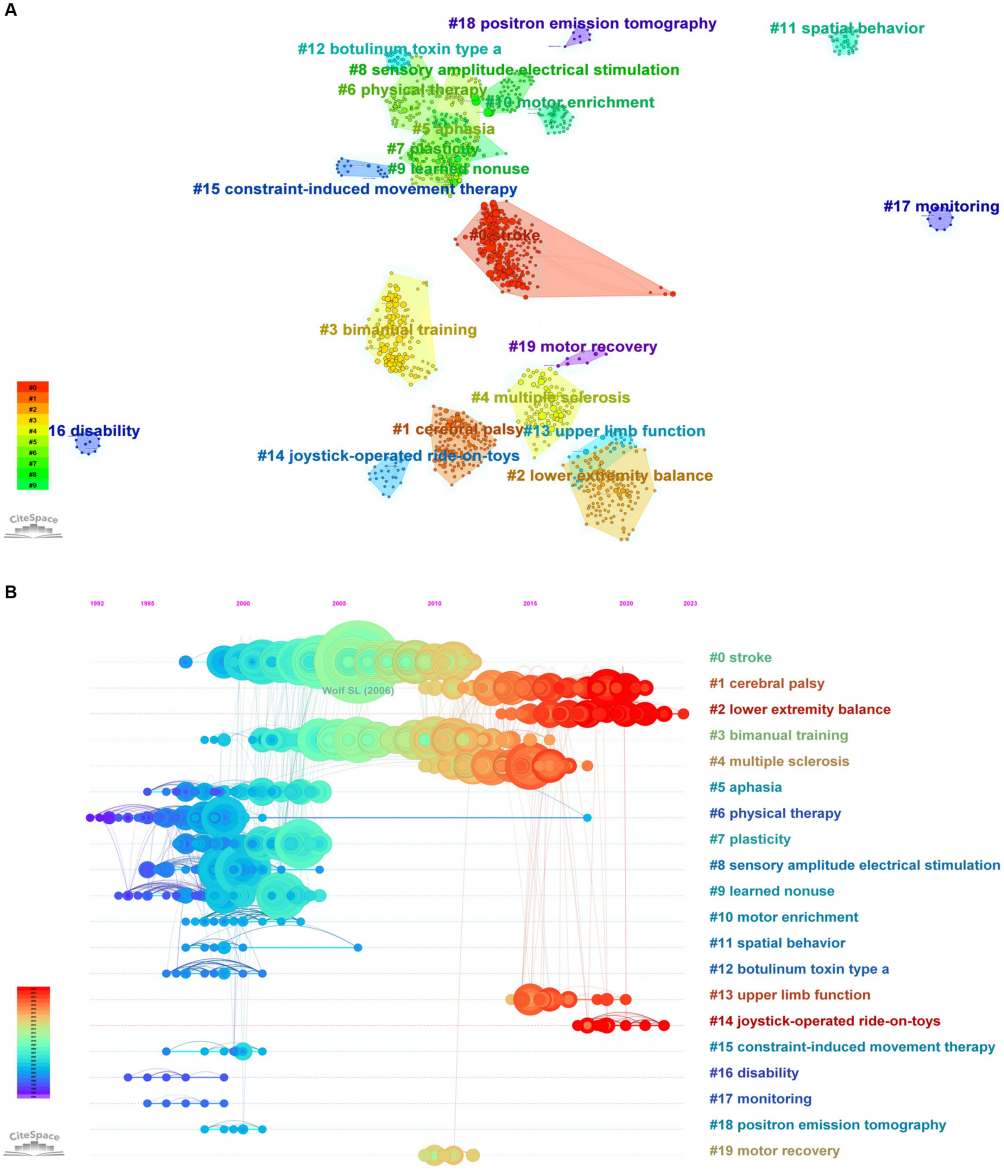

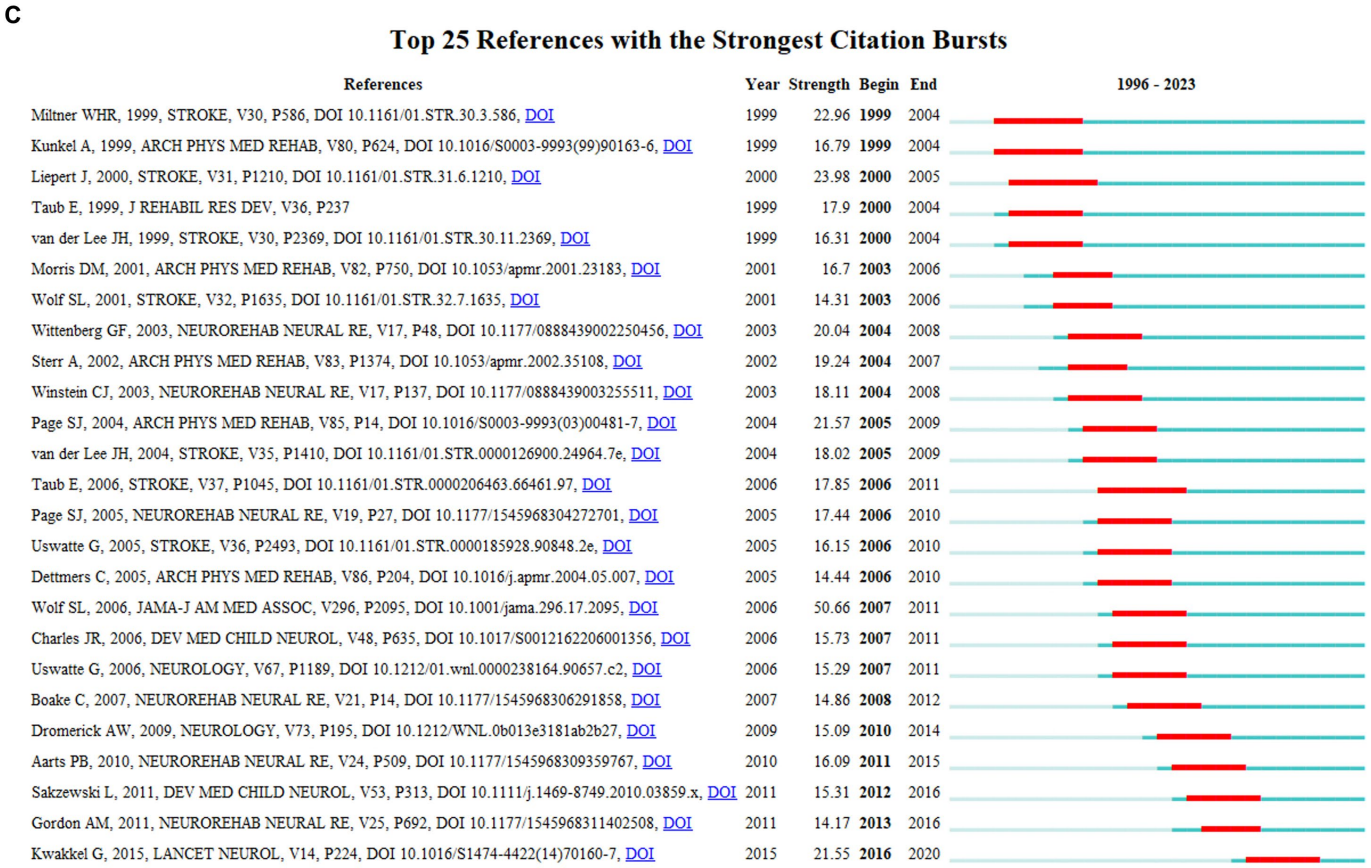
**(A)** Clustering of CIMT co-cited literature in the field of rehabilitation. **(B)** Timeline View of CIMT’s Key Literature in Rehabilitation. **(C)** CIMT in Rehabilitation Key Literature Breakout Chart.

Top 10 most cited literatures are displayed in Table 6. Through examining and evaluating highly cited publications, the areas of interest in research can be revealed. Concurrently, it is possible to identify the areas of research concentration and the development path of CIMT in the field of rehabilitation. The top 10 cited frequency-ranked literature in this field all study stroke, which makes it clear that CIMT for stroke is an enduring research hotspot in this field. In light of the categorization of research methods, they fall into two categories: clinical trials and Meta-analysis. The 1st, 2nd, 3rd, 4th, 5th, 6th, 7th, 8th, and 10th cited frequency items are clinical studies, and the 9th cited frequency item is Meta-analysis. Among them, the cited frequency items 1, 2, 4, 6, 7, 8, and 10 studies used randomized controlled study methods, and the cited frequency items 3 and 5 used non-randomized controlled study methods. The Fugl-Meyer Assessment of Motor Recovery (FMA), the Stroke Impact Scale (SIS), the Wolf Motor Function Test (WMFT), the Motor Activity Log (MAL), the Action Research Arm Test (ARAT), and the Upper Extremity Function Rating Scale were used as testing indexes in the majority of the studies to evaluate the efficacy of the therapy. This was done by comparing changes in the ratings of the visual analogical rating scales over a predetermined period of time. Every clinical study’s findings demonstrated that 2-week CIMT/mCIMT had a better effect on the prognosis of stroke, and the visual analogical rating scale values were significantly different before and after treatment. Subfields that reflect important research hotspots can be shown using cluster analysis based on co-cited literature (24). Twenty clusters in all were created with the traditional LLR technique. Literature clustering can identify the sub-topic directions of the hot research in the field, with the biggest cluster denoted by #0 and the rest by analogy, with the cluster number being inversely proportionate to the cluster size, as shown in Figure 8A. The hot areas of research are stroke, cerebral palsy, lower limb balance, bimanual training, multiple sclerosis, aphasia, physical therapy (transcranial magnetic stimulation), plasticity, sensory amplitude electrical stimulation, learned non-use, motor reinforcement, spatial behavior, botulinum toxin type A, upper limb function (machine learning), joystick ride-on toys (new technology in rehabilitation), CIMT, disability, monitoring, positron emission tomography, exercise rehabilitation. Figure 8B Timeline view of key CIMT literature in Rehabilitation shows that the research trends in this area are lower extremity balance, upper extremity function (machine learning), and joystick riding toys (new technologies in rehabilitation), judging from the brighter color of the clusters. Furthermore, as Figure 8C illustrates, the emerging citations highlight important works that are often cited throughout time, highlighting hotspots and patterns. Out of the 25 emerging citations that the Web of Science database reviewed and found to have the greatest frequency of co-cited literature, the one with the highest mutation intensity was the EXCITE randomized clinical trial using CIMT to treat 222 patients 3–9 months after ischemic stroke, published by Wolf SL in 2006, which resulted in a significant improvement in hand-arm motor function (WMFT, MAL) that lasted for at least 1 year (50.66) (8); followed by the first demonstration by Liepert in 2000 that long-term changes in brain function correlate with treatment-induced improvements in motor rehabilitation after neurological injury. Following chronic phase stroke patients’ CIMT therapy, the afflicted hemisphere’s muscle output area significantly expanded, and recruitment from nearby brain areas was improved. It even lasted until 6 months when the size of motor cortical areas in both hemispheres was almost the same (23.98) (25); in third place, Miltner WHR’s 1999 publication replicating in Germany the results of the US laboratory CIMT to improve WMFT and MAL in chronic stroke patients, demonstrating the general applicability of the intervention (22.96) (26).

**Table 6:** CIMT’s top 10 cited literature frequency rankings in the field of rehabilitation.

Author, Year	Frequency	Title	Journal (IF)	H-index	JCR
Wolf SL, 2006	114	Effect of constraint-induced movement therapy on upper extremity function 3 to 9 months after stroke: the EXCITE randomized clinical trial	Journal of the American Medical Association (120.7)	622	Q1
Page SJ, 2004	49	Efficacy of modified constraint-induced movement therapy in chronic stroke: a single-blinded randomized controlled trial	Archives of physical medicine and rehabilitation (4.3)	169	Q1
Liepert J, 2000	49	Treatment-induced cortical reorganization after stroke in humans	Stroke (6.157)	292	Q1
Taub E, 2006	47	A placebo-controlled trial of constraint-induced movement therapy for upper extremity after stroke	Stroke (6.157)	292	Q1
Miltner WHR, 1999	45	Effects of constraint-induced movement therapy on patients with chronic motor deficits after stroke: a replication	Stroke (6.157)	292	Q1
Wittenberg GF, 2003	42	Constraint-induced therapy in stroke: magnetic-stimulation motor maps and cerebral activation	Neurorehabilitation and neural repair (4.2)	92	Q2
van der Lee JH, 2004	41	Clinimetric properties of the motor activity log for the assessment of arm use in hemiparetic patients	Stroke (6.157)	292	Q1
Page SJ, 2005	41	Modified constraint-induced therapy in acute stroke: a randomized controlled pilot study	Neurorehabilitation and neural repair (4.2)	92	Q2
Kwakkel G, 2015	40	Constraint-induced movement therapy after stroke	Lancet Neurology (48)	259	Q1
Sterr A, 2002	40	Longer versus shorter daily constraint-induced movement therapy of chronic hemiparesis: an exploratory study	Archives of physical medicine and rehabilitation (4.3)	169	Q1

## 6 Discussion

To comprehensively review the literature on constraint-induced movement therapy (CIMT) in the realm of rehabilitation research over the past three decades, this study employs a bibliometric approach. Through visual knowledge mapping, the study delineates the field’s knowledge structure and growth trajectory from various perspectives. The results of the study reveal a significant level of interest among rehabilitation researchers in CIMT. When categorized by research topics, the most prominent area of research is the utilization of CIMT in stroke patients, which constitutes 80.2% of the literature analyzed. Additionally, CIMT applications in cerebral palsy and Parkinson’s disease account for 19.28 and 0.52% of the literature, respectively, while no studies on CIMT application in peripheral neurological disorders were found.

The average year of publishing for academic articles is particularly early in the United States, Germany, the United Kingdom, and Canada, as they are leading research nations. These countries have significantly contributed to the early study of this field, establishing a solid knowledge base for further research. In contrast, China, the second largest producer of research articles, only began to make relevant contributions in 2006. However, China has recently accelerated its development in this field, showing a consistent increase in publications. It is important to note that research trends vary across regions due to different national policies. Taiwan, China has been an early research region in this field, with an annual publication volume of around 3 articles. Mainland China started later but has shown a faster growth rate. In 2023, its growth rate reached 58%, surpassing the US in terms of publication numbers for the first time. However, mainland China’s research output ranks in the bottom 1 of the top 10 countries/regions, indicating that the overall research quality of China’s publications is low and fails to achieve wide dissemination in the field. This highlights the importance of focusing not only on the quantity but also on the quality of research output when aiming for academic prestige and influence in a specific research area. The United States exhibits the highest TLS, demonstrated by its strong research collaborations with Australia and the United Kingdom. The partnership between North America and Europe is notably stronger than its relationships with Asia and Africa. Despite ranking fifth in publishing volume, the United Kingdom boasts the highest ACPP, signifying widespread recognition and citation of its academic research. Notably, North America and Europe collectively represent 50% of the top 10 countries/regions with the highest publication numbers, while 30% are located in East Asia, highlighting these regions as key centers of study in this field.

University of Alabama Birmingham has solidified its position as a leading institution in the field, with notable publications. One such study conducted in Germany replicated a US laboratory experiment using CIMT to enhance upper limb function (WMFT, MAL) in chronic stroke patients, showing lasting improvements up to a 6-month follow-up (26). Emory University stands out for its high TLS and strong collaboration with other institutions. A significant study involving 222 patients 3–9 months post-ischemic stroke demonstrated long-term improvements in arm motor function (WMFT, MAL) through CIMT treatment, which was the first application of a prospective multicenter (EXCITE) randomized controlled clinical trial to confirm the Positive efficacy of CIMT in the treatment of subacute stroke (8). Despite fewer publications, University of Florida ranks highly in academic impact, as evidenced by a study involving 127 patients with upper extremity injuries post-stroke. The study compared CIMT, robotic-assisted treatment, and usual care, showing that robotic-assisted treatment did not significantly improve motor function (FMA, WMFT) after 12 weeks, but did show better results compared to standard care after 36 weeks, highlighting the effectiveness of CIMT in chronic stroke treatment (27). Although China National Taiwan University ranked third in research paper production, but its Average Citation Per Paper (ACPP) was relatively low, suggesting a need for further improvement in research quality. Enhancing collaboration and communication with other leading research groups is crucial. Additionally, it is noteworthy that the average publication year of National Taiwan University’s papers is relatively recent, with a primary focus on comparative clinical efficacy studies of mCIMT.

In the realm of academic publications, among the top 10 writers, respectively, among which Taub E from Alabama Univ, United States, holds the top spot in TC, ACPP, H-index, and TLS in addition to being the most prolific author in the area, His highly cited articles provide evidence that CIMT is effective in improving chronic (>1 year) (26) or subacute (3–9 months) (8) upper extremity function in stroke patients, with long-term benefits. Furthermore, CIMT has been shown to enhance muscle output in the affected hemisphere of chronic stroke patients, leading to increased muscle output and recruitment in adjacent brain regions (28). Even when sustained for up to 6 months, motor cortical areas in both hemispheres can be nearly identical in size (25). This was the first time that CIMT was proposed and demonstrated. This is the first suggestion and illustration that CIMT can enhance the brain’s motor cortex’s plasticity in individuals with chronic stroke. Recent research has explored the efficacy of CIMT in improving motor function in paralyzed lower limbs post-stroke (29, 30), as well as its potential applications in other neurological conditions such as multiple sclerosis (31, 32) and cerebral palsy (33). Taub E’s top ranking in TLS reflects his collaborative efforts with scholars like Uswatte G, Morris DM, Wolf SL, Mark VW, and Miltner WHR from the University of Alabama. In addition, Uswatte G from Alabama University, United States, who ranked second in terms of number of publications, TC, ACPP, H-index, and TLS, is the top-ranked researcher in total link strength. Uswatte G’s research focused on evaluating the accuracy of accelerometry based on CIMT in tracking actual arm movement in subacute stroke patients (34, 35). Notably, Bai YL from Fudan University, representing mainland China, has conducted research on the molecular mechanisms of CIMT to enhance neurological and motor functions in stroke patients. He communicated closely with Ce L and Hu J. This research has focused on areas such as ipsilateral corticospinal tracts (36), sensory-motor cortex and hippocampal synapses (37), phosphorylated extracellularly regulated protein kinase (38), AMPA receptor-dependent synapses in the ipsilateral hemisphere (39), and inhibition of extracellular traps of neutrophils in the ischemic cortex (40). In Taiwan, Lin KC from Natl Taiwan Univ, along with Wu CY and Chen CL, has concentrated on improving motor performance, daily functioning, and quality of life in chronic stroke patients through mCIMT, dCIMT, and constraint-induced therapy with trunk restraints (CIT-TR) in clinical randomized controlled studies (41–43).

Neurorehabilitation and Neural Repair is the most influential journal in the field. Among the most frequently cited papers on a clinical trial of CIMT in chronic stroke patients with aphasia, which showed that CIMT improved several standardized clinical tests, self-ratings, and the effectiveness of communication in patients’ daily lives (44). Stroke is a top-ranked ACPP journal and a Q1 journal with the highest IF. One of its high-profile papers was the first to demonstrate that long-term changes in brain function are associated with improved motor rehabilitation after treatment-induced neurological injury (25). The Q1 journals with the highest IF and H indices are Stroke and Archives of Physical Medicine and Rehabilitation, respectively. This suggests that the articles that have appeared in these two reputable journals have a high academic reference value. You may give these incredibly productive journals priority when submitting research to get published in this subject, and when searching for related literature, you can prefer the proceedings of these highly cited journals.

Highly cited literature often signifies impactful research, with a substantial academic influence, providing insights into key areas of focus within a particular field (45). Therefore, examining the most referenced literature can provide early insights into research trends and directions in the field. The top ten cited works primarily fall into two categories: comparative clinical efficacy studies of CIMT/mCIMT for enhancing upper limb function in stroke patients, and investigations into the molecular mechanisms underlying the efficacy of CIMT in improving upper limb function in stroke patients. Among the comparative clinical efficacy studies of conventional CIMT, an earlier study was that of Miltner et al. who replicated the results of US laboratory CIMT to improve WMFT and MAL in patients with chronic stroke in Germany, demonstrating the general applicability of the intervention (26). Following closely behind, Sterr et al. found that a 6-h CIMT training protocol was more effective than a 3-h protocol in improving motor function (WMFT, MAL) in chronic stroke patients (46). Taub et al. also reported that CIMT significantly improved arm motor deficits (WMFT, MAL) in patients with mild to moderate chronic stroke (47). Notably, Wolf et al. reported the first application of the prospective multicenter, large sample size (EXCITE) randomized controlled clinical trial to confirm the positive efficacy of CIMT in the treatment of subacute stroke (8). CIMT significantly improved arm motor function (WMFT, MAL) in 222 patients 3–9 months after ischemic stroke and persisted for at least 1 year (8). In addition, Peurala et al. in a meta-analysis that included 27 randomized controlled trial studies, found that 60–72 h of CIMT exercises over a two-week period produced better mobility. And only 30 h of CIMT exercises in 3 weeks showed improvement (48).

In a comparative study of the clinical efficacy of mCIMT, the most frequently cited is Page et al. conducted a mCIMT 3 times/week for 10 weeks, with the less affected arm being restricted 5 days/week for 5 h, and the findings that mCIMT improved the function of the more affected arm in chronic stroke patients and the use, further confirming that repetitive, task-specific exercises are critical for regaining function, whereas the intensity of the exercise program is less important (49). For acute stroke patients, Page et al. found that mCIMT improved the use of the affected arm, MAL, Fugl-Meyer, and ARAT, and the ability of the patient to perform valuable activities again, compared with the efficacy of conventional rehabilitation for the treatment of upper extremity hemiparesis in acute stroke patients less than 14 days post-stroke (50). In addition, a review published by Kwakkel et al. describes the current evidence regarding, original CIMT and mCIMT. The beneficial effects of the original and mCIMT types on motor function, arm-hand activity, and self-reported arm-hand function in daily life, immediately after treatment, and at long-term follow-up were summarized (51). Of note, the optimal timing, dose, and training intensity of CIMT/mCIMT interventions in acute and chronic stroke patients are not yet defined.

Among the studies on the molecular mechanisms of CIMT efficacy, Liepert J and Bauder H (2000) were the first to propose and demonstrate that CIMT improves plasticity in the motor cortex of the brain in chronic stroke patients. Their 12-day CIMT intervention with 13 chronic stroke patients compared the cortical motor output areas of the hand muscles on both sides before and after treatment to evaluate the reorganization of the motor cortex following effective rehabilitation therapy in stroke patients. The results showed a significant enlargement of the muscle output area in the affected hemisphere and increased recruitment from neighboring brain regions in chronic stroke patients. These changes were observed to persist for up to 6 months, with the size of motor cortical areas in both hemispheres becoming nearly identical (25). Additionally, Wittenberg et al. utilized transcranial magnetic stimulation to map the motor cortex and positron emission tomography to assess changes in motor task-related activation as a result of CIMT intervention. Their findings indicated that CIMT treatment led to a significant decrease in brain activation during the motor task, along with an expansion of the motor map in the affected hemisphere. This suggests an enhanced capacity of the upper motor neurons to generate movement (52). The underlying mechanisms driving both the CIMT/mCIMT are still poorly understood, but results from kinesiological studies suggest that improvements are largely based on post-stroke adaptation through learning to optimize the use of intact end-effector for some autonomous motor control of wrist and finger extensor muscles (51). These influential publications have made a substantial academic contribution and have been instrumental in pushing the field forward. Continued analysis of these publications will enhance our comprehension of the present state and future trajectory of the field, ultimately bolstering our research capabilities and the quality of our research outcomes.

In recent years, there have been several studies comparing CIMT to other therapies, for example, to proprioceptive-based training (13), compared to an unconstrained task-oriented training group (53), CIMT has been shown to improve motor function and ADLs and can be used in combination with other therapies. For example, in combination with virtual reality training (54) and botulinum toxin A injections (55), visual biofeedback training (56), combined with mirror therapy (57), which resulted in better improvements in upper limb motor function, ADLs, grasping and pad-pinch function. It can be assumed that CIMT, in combination with other treatments, produces additional results. This suggests that we need to choose the most appropriate treatment for the patient based on their condition and needs. It is noteworthy that the use of trunk surrogate methods may hinder the upper extremity’s long-term functional rehabilitation. CIMT can be augmented with trunk restraints. It has been demonstrated that in terms of upper extremity motor function, ADLs, and use of the hemiplegic upper extremity, CIMT in conjunction with trunk restraint is much better than CIMT alone (12, 58). Enhancing upper limb motor performance may also be possible by combining CIMT with transcranial magnetic stimulation (TMS) or transcranial direct current stimulation (tDCS) (59–61). A major field of study is robot-based rehabilitation, which offers precision control and real-time patient monitoring to achieve effective rehabilitation. A research comparing CIMT with robot-assisted treatment shown that while both enhanced patient performance, there was no discernible change in motor function across the groups (62). Nevertheless, compared to robot-assisted therapy alone, studies that combined CI therapy with robot-assisted therapy demonstrated a decrease in compensatory trunk movements during activities and a greater improvement in motor function and ADLs (63, 64). In the aftermath of the new coronavirus era, home rehabilitation and telemedicine have become hot study issues. Studies have shown that CIMT in conjunction with telemedicine and home rehabilitation using a gaming approach can have efficacy comparable to clinic-based rehabilitation (65, 66). For stroke patients who require long-term rehabilitation, it is particularly practical and effective.

CIMT for the lower extremities in stroke patients is a modification of the original CIMT for the upper extremit (30). In lower extremity CIMT, the number of exercise tasks, rather than the duration of the exercises, might be crucial to the functional recovery process. As a result, using the mCIMT, which prioritizes repeat count over length, could be more practical (67). Numerous studies have demonstrated that CIMT considerably enhances patients’ walking speed, lower limb motor function, and balance (68). In addition, treatments for aphasia should also incorporate behavioral and communicative correlates of interactions throughout therapy, as well as a thorough training program tailored to the individual’s communication requirements and abilities. These principles have been adopted by new treatments (6). CIMT can be used to treat acute post-stroke aphasia (69) and to improve chronic aphasia (>1 year) (70). The benefits derived from it may persist long after treatment has ended (71). In addition, CIMT may improve depressive symptoms (72) and unilateral spatial neglect (16). Impairment of hand function is a major dysfunction in children with cerebral palsy, and children often tend to use the healthy side more in daily life, leading to “learned disuse.” CIMT and mCIMT are equally useful for improving spasticity and cerebral palsy children’s upper limb motor function, with benefits lasting long (14, 33, 73).

Keywords and key literature co-occurrence and clustering analyses showed that CIMT, stroke rehabilitation, upper limb function (machine learning), lower limb gait balance, randomized controlled trials, physical therapy techniques (transcranial magnetic stimulation and sensory amplitude electrical stimulation), primary motor cortex plasticity, lateral dominance (spatial behavior), cerebrovascular accidents, activities of daily living, hand function, disability, functional restoration, cerebral palsy, bilateral arm training, aphasia, learned disuse, botulinum toxin type A, and joystick riding toys (new technologies in rehabilitation) are the main research hotspot keywords in this field. The clustering timeline graphs of keywords and key literature can be observed that the clustering labels of #0 activities of daily living, #2 hand function, #5 gait, #6 disability, and #2 lower extremity balance, #13 upper extremity function, and #14 joystick riding toys, respectively, are still evolving, which to a certain extent reflects the current research hotspots. Research hotspots that have received a lot of attention over time are known as burst keywords. Keywords that have exploded in the field in recent years include motor function, human, transcranial direct current stimulation, and systematic review. These research hotspots offer insightful information for next studies in addition to reflecting present trends. Specifically the main research hotspots are the clinical efficacy of CIMT combined with other therapies (transcranial direct current stimulation, botulinum toxin type a, virtual reality, mirror therapy, and robotic assistance) to improve the function of upper extremity hemiparesis in patients with stroke, the mechanism of CIMT to improve the plasticity of the motor cortex through electrophysiological and imaging methods, and improvement of lower limb gait balance function in stroke patients and aphasia applications. In the future, we can further delve into the optimal intervention time and dosage of CIMT for different stages of stroke and the exploratory application of CIMT in new environments, such as robotic-assisted, telemedicine, and home-based rehabilitation, with the goals of enhancing the effectiveness of the rehabilitation program and offering practical assistance for the stroke rehabilitation industry.

### 6.1 Limitations of the study

This research may have missed excellent literature from other databases in the area or in other languages because it only included studies of literature in the English language from the WOS database’s core dataset. It also has certain limitations when it comes to literature retrieval. That being said, it is crucial to stress that the WoSCC database is generally accepted as the most extensively used database for bibliometric research (74–76).

## 7 Conclusion

This work offers a fresh viewpoint for a rapid comprehension of CIMT in rehabilitation as it is the first bibliometric and visualisation analysis of CIMT in rehabilitation research during the previous 30 years from many angles. The current state of research suggests that CIMT in rehabilitation research still has vast potential for growth. The most influential countries, institutions, journals, and authors are the United States, Alabama Univ, Neurorehabilitation and Neural Repair, and Taub E. The research hotspot keywords are CIMT, stroke rehabilitation, upper extremity function (machine learning), lower extremity gait balance, randomized controlled trials, physical therapy techniques (transcranial magnetic stimulation and sensory amplitude electrical stimulation), primary motor cortex plasticity, lateral dominance (spatial behavior), cerebrovascular accidents, activities of daily living, hand function, disability, functional restoration, bimanual training, aphasia, learned disuse, botulinum toxin type A, and joystick ride-on toys (new rehabilitation technology). These hot keywords reflect, to some extent, the development trend and cutting-edge research direction of the field. However, CIMT still faces many opportunities and challenges in rehabilitation research, including the clinical efficacy of CIMT combined with other therapies (botulinum toxin type A, transcranial direct current stimulation, virtual reality, mirror therapy, robotic-assisted) to enhance the functionality of upper limb hemiparesis in stroke patients, the investigation of the mechanism of CIMT to improve motor cortex plasticity through electrophysiological and imaging methods, and improvement of lower limb gait balance function in stroke patients and aphasia applications. Meanwhile, future studies should focus on an in-depth exploration of the optimal intervention time and dose of CIMT for different stages of stroke CIMT in new environments such as robot-assisted, telemedicine, and home rehabilitation. Overall, this research offers a comprehensive and well-structured overview of the extensive and intricate literature on CIMT in the field of rehabilitation. It is given in the form of a knowledge map. This facilitates the comprehension of the discipline’s history and future prospects by academics, hence promoting further scholarly investigation.

## Data availability statement

The original contributions presented in the study are included in the article/Supplementary material, further inquiries can be directed to the corresponding author.

## Author contributions

JX: Conceptualization, Software, Writing – original draft, Writing – review & editing. MC: Formal analysis, Writing – review & editing. XW: Investigation, Writing – review & editing. ZC: Funding acquisition, Supervision, Writing – review & editing. YW: Funding acquisition, Supervision, Writing – review & editing. XL: Funding acquisition, Project administration, Writing – review & editing.
