# Supplementary material for: Global research hotspots and trends in constraint-induced movement therapy in rehabilitation over the past 30 years: a bibliometric and visualization study
Source: Front Neurol. 2024 Jun 14;15:1375855. doi: 10.3389/fneur.2024.1375855 (PMC11211381; doi:10.3389/fneur.2024.1375855)
Supplement: Supplementary file 1 [file Table_1.DOCX]

Supplementary table 1

Table S1. Search strategy of Web of science Collection.

| #1 | TS=(Constraint-induced movement therapy OR Constraint induced therapy OR Constraint induced movement therapy OR Intensive exercise therapy OR Forced use) |
| --- | --- |
| #2 | TS=(Rehabilitation OR Habilitation) |
| #3 | #1 and #2 |
